# Do Not Worry That Generative AI May Compromise Human Creativity or Intelligence in the Future: It Already Has

**DOI:** 10.3390/jintelligence12070069

**Published:** 2024-07-19

**Authors:** Robert J. Sternberg

**Affiliations:** Department of Psychology, Cornell University, Ithaca, NY 14853, USA; rjs487@cornell.edu

**Keywords:** generative AI, intelligence, creativity, wisdom, self-consciousness

## Abstract

Technology alters both perceptions of human intelligence and creativity and the actual processes of intelligence and creativity. Skills that were once important for human intelligence, for example, computational ones, no longer hold anywhere near the same importance they did before the age of computers. The advantage of computers is that they may lead us to focus on what we believe to be more important things than what they have replaced. In the case of penmanship, spelling, or arithmetic computation, such an argument could bear fruit. But in the case of human creativity, the loss of creative skills and attitudes may be a long-term loss to humanity. Generative AI is replicative. It can recombine and re-sort ideas, but it is not clear that it will generate the kinds of paradigm-breaking ideas the world needs right now to solve the serious problems that confront it, such as global climate change, pollution, violence, increasing income disparities, and creeping autocracy.

## 1. Introduction

Generative AI can accomplish many positive things. For those whose writing skills are less than fully developed, generative AI can improve the quality of their writing. Generative AI also can help one generate ideas for writing or speaking, and it can evaluate one’s ideas as well. But the focus in this article is not on what generative AI does *for* us, but rather on what it does *to* us. The greatest worry in these times of generative AI is not that it may compromise human creativity or intelligence, but that it already has.

People have always been afraid that technology would deprive humanity of its ability to adapt and perhaps ultimately even replace humanity. On the positive side, humanity still exists. But on the negative side, changes that occur slowly are often missed because they occur on a slippery slope. The world has never been exposed, in quite so widespread and systematic a way, to the amount of misinformation and disinformation that the internet can and does provide. For those who view this as a small price to pay, one might note that the author’s own country, the United States, has not been as polarized politically and ideologically as it is now since the U.S. Civil War of 1861–1865 (Manchester 2018; Paisley 2016). And it is not only the United States that is so divided. Other countries are experiencing similar polarization (Carothers and O’Donohue 2019). Maybe misinformation and disinformation, including that generated by AI, truly are changing the world in ways that are highly problematic. What might be going on? Consider five issues.

### 1.1. Self-Consciousness

First is the matter of self-consciousness. The greatest danger of generative AI is not the takeover but a takeover that one does not even realize is happening. Consider an analogy to parasitic illness. Certain kinds of parasites are brain-altering, but the organisms affected by the parasites serve the parasites without even realizing they are doing so (Cranage 2023). Analogously, generative AI is already taking over people’s minds and leading people to make bad political and other self-defeating choices, believing that they are operating of their own free will (Ecker et al. 2022). Disinformation and misinformation from generative AI are leaving people unable even to know, in an adequately reflective way, what to say, think, and do (Ryan-Mosley 2023).

### 1.2. Exercise

Second is the matter of exercise. Almost all human functions obey the law of “use it or lose it” (Smith 2023). People lose muscle mass if they do not use their muscles, but they can also lose their ability to compute, handwrite, spell, or write text if computers do it for them. And computers are doing so. As Flynn (2012, 2016) has pointed out, the main way to enhance intelligence is to exercise it by confronting difficult adaptive challenges. And Vygotsky ([1962] 2012, 1978) pointed out that the best challenges are ones that are just beyond the cognitive level at which one is currently functioning.

### 1.3. Intellectual Ownership

Third is the matter of intellectual ownership. In the past, if a student paid someone to write a paper for them, they knew they were in trouble. But students are coming to view generative AI products as “their” projects, because they provided the prompts to the AI. So, they are losing their sense of guilt and shame while they are claiming intellectual ownership of products that are not truly theirs. They see their query to the AI as the basis of the creativity and intelligence of the work. This is more of a problem than with spelling (e.g., through auto-correct on computers) or computation (e.g., through programs to perform data analysis) because whereas spelling and computation have “right vs. wrong” answers, writing that is creative does not have a single “correct” solution. As they lose their abilities to think creatively and intelligently, they may believe they are increasing them.

### 1.4. Detectability

Fourth is the matter of detectability. Generative AI has reached the point where there are no adequate programs for detecting its use. The software that exists, at least at present, is unreliable in its detection, with numerous false alarms and misses. So, there is a great incentive to use it if others are using it and one sees that there are no consequences, even if its use is forbidden. 

### 1.5. Hidden Purposes

Fifth and finally is the matter of whether generative AI has behind it purposes of which the user is unaware. If the programmers have certain goals that they want to accomplish to lead people to think one way or another, or if the programmers are being compensated by clients, such as governments, to program in certain ways that benefit the governments, people may become unwitting dupes of masters of whom they are unaware, and whose intentions are unknown to them. Much of what is presented on the internet as objective is anything but objective and may be software-generated to accomplish particular goals that are hidden in the programming that the end-user cannot see.

### 1.6. The Bottom Line

The bottom line is that technology is moving much faster than our wisdom in monitoring the uses of the technology, with results that may be, in the long as well as the short term, destructive to humanity and its true autonomy, as opposed to its perception of its own autonomy.

A cartoon by Dave Whamond, ©2024 Cagle Cartoons, Inc., shows two robots and a bunch of young people in front of them (https://cagle.com/dave-whamond/, accessed on 1 July 2024). One robot says to the other: “Initiate Phase One of the robot takeover”. The other robot, seeing all of the young people staring at their cellphones, replies: “Y’know, Frank, I don’t think we need to bother…”.

That is a theme of this article—that the generative AI takeover is not a worry for the future. It already has already occurred. There are different views, ranging from positive to negative, and some believe that a crisis is at hand (Edsel 2024). It is not clear that there is a single answer: AI could be programmed to be a personal digital assistant that makes one’s life easier or to autonomously choose human targets to be eliminated, based on the AI’s judgment of whether the targets are dangerous. It is worrisome that much of the strongest criticism of AI, and particularly of generative AI, comes from employees of companies that produce generative AI; it is even more worrisome that the companies have tried to suppress warnings of danger, suggesting, at least to some people, that the companies have something to hide (Lessig 2024). If there is nothing to worry about, why make life difficult for those who issue warnings? 

The purpose of this essay is not to discuss whether AI, in general, or generative AI, in particular, will be good or bad for humanity. It will probably be good in some ways and bad in others, as is true with so many innovations. But at this point, the answer is up to us, and if those who produce generative AI are too greedy to care and legislators too busy raking in votes and money to be bothered, we probably will get what we deserve, as we are getting with global climate change, pollution, school violence, and so many other issues that seem to elude the attention of legislators who prefer showmanship and showboating to legislating. Rather, as this is an essay based on psychological research, the focus will be on the risks that generative AI poses to us as humans and thinkers, regardless of whether we use AI for positive, negative, or both types of purposes.

The greatest problem is that much of AI is what has been referred to as *para-transformational* for society (Sternberg and Dashtaki 2024; Sternberg et al. forthcoming). Para-transformations are ones that typically have both positive and negative aspects, but at a given time, it is not clear whether the positive or negative ones will predominate in the long term.

The enemy initiating the takeover is not really generative AI. It is you and I, and almost everyone else, too. U.S. teenagers spend an average of 7 h 22 min per day on screens (Duarte 2023). What are they doing all this time? What are the author’s 13-year-old triplets doing with their time on screens?

There are three issues, in particular, that will be discussed in this article, elaborating on the issues raised at the beginning of the article.

1. **Skills that are not used tend to become degraded, following the exercise principle of “Use it or lose it.” If generative AI does our creative work for us, we risk losing our creativity because we do not exercise it.** Thus, we may show a sort of reverse Flynn (2012, 2016) effect, whereby creativity decreases not through circumstances beyond our control but rather through circumstances we could control but choose not to.
2. Worse, **we may come to believe that products of middling or even mediocre creativity are better than they are** simply because we do not want to be bothered to be creative or because we have lost our ability or drive to be creative.
3. Still worse, **we may come to believe that what the generative AI produces is our production.** How many millions of students and workers right now are handing in generative AI work and claiming sole authorship without citing the use of generative AI? It used to be that if you hired someone to write a paper for you while you were a college student, you were likely to be expelled, as was Henry Ford II when he paid another student to write a paper for him while he was a student at Yale (DeMott 1987). He knew the paper was not his. Do today’s students know the same? After all, when they do calculations on a computer or use a word-processing program to correct their spelling or grammar, they usually do not credit the software.

## 2. Use It or Lose It

Many readers will have heard the expression “Use it or lose it” as it applies to physical exercise. If one fails to exercise sufficiently and properly, one risks losing various kinds of physical functions. Exercise becomes especially important with age, as there is a tendency to lose muscle mass and flexibility with age (Volpi et al. 2004). “Use it or lose it” applies to mental function as well as to physical function (Fauth and Norton 2024; Harrison et al. 2015; Henderson 2014; Scarmeas and Stern 2003). The principle applies not only to individuals, but also to societies. How many of us in post-industrial societies have the hunting and gathering skills that once would have been necessary for survival? As the skills became less important for environmental adaptation, parents and teachers stopped teaching children how to do these things.

The example of hunting is of a skill long gone for most people. But there are skills that have much more recently begun to disappear. Between 2016 and 2021, enrollments in foreign languages saw their steepest decline ever (Nietzel 2023; Quinn 2023). The reasons are undoubtedly varied and complex. Nevertheless, in 2023, West Virginia University shocked many when it eliminated all of its degree offerings in foreign languages. Only 7% of the college students in the United States study a foreign language, and only 20% of the relevant school-age population (Rampe 2023). For many students, the feeling is, why bother? *There are now so many different AI-based foreign-language programs that one can enter fairly long foreign-language texts into them and get near-immediate translations.* Or to say it a different way: “*Erilaisia tekoälypohjaisia vieraiden kielten ohjelmia on nykyään niin paljon, että niihin voi syöttää melko pitkiä vieraskielisiä tekstejä ja saada lähes välittömiä käännöksiä*”, which is the same sentence as the previous one, translated by Google Translate in less than a second into Finnish, of which the author of this article speaks not a word. Why learn a foreign language when software can do the work for you, or at least, much of it?

Well, there are good reasons to learn a foreign language, of course. When you want to have a conversation with people in a foreign language, how good will the conversation be if you keep using a translator that may or may not be accurate? If someone from a foreign country comes here and you need to get to know them, how well will the automatic translator work in helping them get to know you, or you get to know them? With increasing globalization, one might have expected that people in the United States would want to increase their foreign-language capabilities, but the opposite has happened. Perhaps some expect others to learn English, but many will not do so, any more than the people in English-speaking countries will learn their foreign language. And if it is a second language for them, you might have had a much better conversation if you were fluent and spoke with them in their own language.

Spelling has become less important than it used to be because one no longer has to look up every word one does not know how to spell. Word-processing programs almost all have spell-checkers that will auto-correct spelling errors. Arithmetic computation has become less important because calculators and computers have taken up doing much of the computation. The author’s middle-school children are encouraged to use calculators, but their long division is already rusty, and when they have to perform a calculation, even a fairly simple one, they turn to a calculator. Handwriting? Who cares? Computers print for us, but my middle-school children have not been taught cursive in their school, so they better have a computer to print for them. And now, with generative AI, why learn how to write a paper when one of the generative AI programs can write it for you?

Oddly, the problem that generative AI creates is not a new one but a successor to a problem that some have felt that the schools created long ago (DeBono 1973, 2015; Robinson and Aronica 2016), namely, that schools, however well-meaning they may be, may damage creativity by forcing children to think along set paths (Sternberg 2010, 2018). In autocracies and corrupted democracies, things can be worse, as schools may teach students propaganda rather than the legitimate educational content that would help the students learn to think rather than suppress their thinking. Sternberg (1985) made this argument in terms of the heavy focus of schools on memory and analytical skills. He argued that students’ creative skills are suppressed by a focus on what will be tested by standardized tests, so that students cease to be creative not because they cannot be but because they have no incentive to be; as a result, they lose their creativity. In that case, generative AI may be, ironically, just finishing up the job that many schools started.

If people use fewer and fewer of their skills, or at least potential skills, what will replace the skills they are not using? Will they develop different skills that are more relevant to the world of today or of tomorrow? Or will their knowledge and skill base decrease because they have generative or other AI to do what they otherwise might have done?

## 3. Loss of Function Is Not Necessarily Accompanied by Regret: It May Not Even Be Noticed

There are very few adults in the developed world who have been trained to hunt, gather, and generally, forage. If, suddenly, people’s access to food were cut off and they had to fend for themselves, many people would be at a loss. People who get lost in a large forest, desert, or mountainous area may quickly become aware of their inability to fend for themselves, or at least to adapt to circumstances that were routine matters of adaptation for their ancestors. But in a post-industrial society, few people go around bemoaning their failure to acquire the skills that were routine for their ancestors. The same obliviousness to loss applies to languages as it applies to humans: Languages go extinct because no one speaks them, so there is no one to bemoan their loss except linguists, some historians, and perhaps some nostalgic descendants of those who spoke the languages. But if there is no one with whom to communicate, one’s incentive to learn the language may be reduced considerably.

Lubart and Sternberg (1995) suggested a cohort effect whereby people tend to view as freshly creative works that are creative for their own time. For example, at the advent of the age of Impressionism, Impressionist work was considered creative. Today, it is still regarded as creative for that time, but an Impressionist artist today has largely missed their optimal time to be judged as creative. The same applies to almost any field, of course. The behaviorist work of B. F. Skinner (e.g., Skinner 1953) was extraordinarily impactful for its time. Even when I started college, Skinner was required reading and many departments of psychology were behaviorist, frowning upon work that focused on anything but overt behavior. Today, many of my students do not know who Skinner was, and even those who do are unlikely to be conducting purely behaviorist learning experiments. The psychology department in which I spent many years, like many psychology departments, once had an entire division devoted to animal learning—mostly behaviorist—but no longer does. The children’s classics that made up the core of my early education are difficult to find today. My own children, like so many others, have little interest in reading them.

The reason this cohort effect matters is that it is not only the type of creativity that varies with time but also levels of creativity. Societies pass through phases (Simonton 1984, 1994) with regard to creativity. Simonton has suggested a number of factors that might influence creativity in a society, such as the concentration of creative individuals in cities; however, in times (or places) when (or where) creative productivity is lower, people may be fully engaged and not spend their time bemoaning what later or elsewhere may be viewed by others as their loss of creativity.

A serious risk of generative AI is that as it decreases creativity, people may not even notice. Rather, they may achieve an adaptation level to the circumstances of their lives (Helson 1948). If people, more and more, leave it to generative AI to do their creative thinking for them, they may not feel any sense of loss, any more than people who use calculators feel a loss of their computing skills or people who use computers feel a loss of their penmanship skills, or even earlier, people who used ballpoint pens felt a loss of the use of quills in writing.

Adaptation levels occur in all aspects of human behavior, as Helson (1948) observed. In science, revolutionary ideas, when they are first proposed, are often rejected and even scorned. The classic example is perhaps Ignaz Semmelweis, who recommended that physicians wash their hands in between their treatment of patients. His ideas not only failed to be accepted but they also were largely derided by other physicians of his time (Tyagi and Barwal 2020).

Thomas Kuhn (2012), in his classic work on the structure of scientific revolutions, pointed out that those who do highly creative, even revolutionary science, are (a) atypical in the scientific community, (b) often unappreciated or derided by their colleagues, and (c) as a result, often have trouble getting their work accepted. The most gifted creators are individuals who excel in recognizing what problems are even worth solving (Getzels 1979; Getzels and Csikszentmihalyi 1976; Zuckerman 1983). An examination of the hundred statistically most eminent behavioral and brain scientists of the twentieth century revealed that they excelled in finding problems that set them apart from others in their field (Sternberg et al. 2016). But those who are not setting the agenda do not feel they are uncreative: They see the agenda-setters as the problem, not themselves, which is why the agenda-setters need to defy the crowd and the zeitgeist (Sternberg 2018).

The larger issue is that people are creative in large part due to their willingness to defy the crowd (Sternberg and Lubart 1995) and, beyond that, to defy themselves and the sociocultural zeitgeist (Sternberg 2018). Thus, creative people see how other people think, and if they find great uniformity in the others’ thinking, question whether the uniformity indicates that something is indeed wrong—that they have merely accepted what they were told rather than questioning it (Tulving 1972). But there are numerous reasons why they might not ask such questions, and thus, might not be creative.

First, many individuals, especially college students, tend to accept what they read on the internet as true, regardless of whether it is true or not (Flanigan and Metzger 2000; Rand and Sirlin 2022; Walker 2020). Adults tend to carefully curate what they read, but this curation is extremely tilted toward myside bias (Butler 2017; Stanovich and West 2000; Stanovich 2009, 2021; Stanovich et al. 2013). They choose what to read in accordance with their already established beliefs, and seeing contrary views may actually strengthen their original bias. Good reasoners often use their reasoning not to improve the accuracy of their beliefs but rather to strengthen their preexisting beliefs, whatever they are (Anderson et al. 1980; Billig 1996; Guyote and Sternberg 1981; Mercier and Sperber 2011). Thus, if generative AI produces text, readers have trouble distinguishing AI-generated from human-generated information, and AI is particularly good at generating disinformation (Williams 2023). In particular, Williams (2023) stated: “Disinformation generated by AI may be more convincing than disinformation written by humans, a new study suggests”. Spitale et al. (2023) stated that “in comparison with humans, [generative AI] can produce accurate information that is easier to understand, but it can also produce more compelling disinformation. We also show that humans cannot distinguish between tweets generated by GPT-3 and written by real Twitter users. In comparison with humans, it can produce accurate information that is easier to understand, but it can also produce more compelling disinformation. We also show that humans cannot distinguish between tweets generated by GPT-3 and written by real Twitter users”.

Second, students may be discouraged in school from asking too many questions or any questions at all. It is hard to get through school without having multiple teachers who discourage thoughtful questioning, or sometimes, any questioning that is nontrivial (Spear and Sternberg 1987; Sternberg and Spear-Swerling 1999; Sternberg 1986). And oddly, some teachers, when they ask questions, even ones that require thoughtful reflection, tend to answer their own questions quickly (Rowe 1986). Skilled teachers, however, wait for students to answer questions and they teach students to ask their own questions (Rothstein and Santana 2011). But the lesson often is that one is expected to defer to authority rather than question it. Almost the entire process of standardized testing is, in effect, a lesson in accepting the framing of questions and answers given by authorities (McCarthy and Blake 2017). One comes to accept framing, whether from a human teacher or a supposed AI teacher. 

Third, more and more national governments are becoming autocratic (Albright 2018; Applebaum 2021; Dictatorship Countries 2024; Levitsky and Ziblatt 2018). Dictatorships encourage a closed-mindedness that supports them (Kruglanski 2013). Those who are open to other ideas or who, worse, promote them, may find themselves silenced, imprisoned, or killed. People often are attracted to toxic leaders (Lipman-Blumen 2006), and those leaders enforce codes of thinking and behavior, much as in *1984* (Orwell 1950).

Generative AI can be an extremely effective tool for autocratic governments. Merely training the AI on large numbers of samples that espouse and support the government’s dogma, no matter how silly or ridiculous, can reinforce these views widely (Briggs and Cross 2024). Countries such as China that control internet content and that conduct mass surveillance on a national scale (Buckley and Mozur 2019; Chin and Bürge 2017), or, like Russia, that imprison people for any statement anywhere that is taken to threaten the established regime, can destroy creativity through AI trained to teach only the authorities’ ways of thinking. Other AI can be trained to identify anyone who shows any sign of thinking other than the government-approved way. Moreover, generative AI can spread conspiracy theories, which people then pick up on (Rogers and Mithani 2021). Eventually, the people may conclude that the truth is unknowable, and they should simply accept what the government tells them. Generative AI thus can serve a useful role in both thought and behavioral suppression, as it can be programmed in ways that lead people to believe that they are receiving truly artificial intelligence, whereas what they are receiving is human intelligence artificially filtered for cynical and often nefarious purposes.

Fourth, as has been known for a long time, people have a tendency to conform to group norms (Hovland and Janis 1959; Hovland et al. 1953). They want to belong (Baumeister and Leary 1995), and thereby fulfill a fundamental human need. They often conform even when they realize that conforming is leading them to say something that is not and could not be true (Asch 1956). If what they hear from authorities and others around them is uniform but unverified, they will tend to go with it, and often not even want to know if it is true because such a quest for truth might upset their sense of belonging. Autocrats in Germany, Rwanda, Russia, and other places have used such desires not to know the truth to conduct genocide and not be called to task for it—at least until too late.

## 4. Acceptance of Mediocrity in Creativity

Why would one accept humdrum, paradigm-preserving creativity—as often produced by generative AI—even more than one would accept outstanding creativity? There are several reasons.

First, humdrum creativity tends to be either replicative or a small increment beyond where things currently are in a field or domain of endeavor (Sternberg et al. 2002). Such creativity is usually nonthreatening. It does not upend anyone’s way of thinking or doing things. One can maintain one’s current belief system and action repertoire without having them challenged.

Second, humdrum creativity is often easier to understand and more palatable than creativity that blazes a new path. One can apply what Piaget (1950, 1952) referred to as assimilation—understanding new ideas in terms of one’s existing schemas —rather than accommodation—creating new cognitive schemas for understanding the ideas. People often prefer material that is easy to absorb, especially when faced with an innovation that they are afraid they will not understand and that they worry will leave behind. The creativity of most generative AI is palatable because, by its nature, it is a reassortment of existing ideas that are recombined from its database.

Third, the social and even economic costs of following humdrum creative ideas tend to be lesser than the costs of following more innovative ideas. If things go wrong, with humdrum ideas, one is pretty much where one was before. One has invested little in changing one’s ways. But with highly innovative ideas, one may have to make changes that, if they do not work out, are costly to one’s self-esteem and social position. Why not go with safe ideas with less downside rather than with highly innovative ideas that may require a great deal of change and introduce a substantial measure of risk?

Finally, humdrum creativity is easier to create than more serious creativity. It may require less knowledge, and it typically requires less of a creative leap. One has a powerful incentive to use and generate it because so little is at stake.

In short, the kind of recombinative creativity that generative AI is capable of producing may be much more understandable than the more radical creativity that can be humanly created. The most popular authors, at any given time, tend not to be those who are hardest to understand but rather those who can be understood without undue effort—more like James Patterson than James Joyce.

## 5. The Matter of Intellectual Ownership

As I write, an unknown number of scientific papers are being written by generative AI (Maiberg 2024). I say “an unknown number” because, at present, there is no definitive way of detecting AI-generated text. The plagiarism-detection program used by the author’s own university has an AI-generation detector but faculty at the university are not allowed to use it because its reliability is so low. And as one would predict, as soon as an AI detector is offered to the public, so are tips for fooling AI detectors (e.g., Juhasz 2023). The author recently submitted an article to a scientific journal, and for the first time, encountered on the website a box asking me whether the author had used AI in the production of the paper. It did not forbid the use of AI; rather, it merely required acknowledgment of its use. The cat-and-mouse detection game will become more sophisticated, but it is unlikely to end, at least in the short term, in anything other than a stalemate.

Why would people use generative AI to produce papers? That is an easy question to answer, unfortunately. Such papers can be produced quickly, cheaply, and with no currently reliable means of detection. And one may have no ideas of one’s own, so AI is the alternative to not producing at all and possibly risking one’s academic average or job.

Unlike in the case of Henry Ford II, for many people, it is at least ambiguous whether the use of generative AI constitutes plagiarism or the appropriation of someone else’s ideas (Jacob 2023). My intention here is not to review the arguments but rather merely to point out that if one is eager to believe that the work is one’s own, one can find plenty of company, at least on the internet, to support one’s point of view. Often, people merely believe what they want to believe (Mercier and Sperber 2011). 

If authors are allowed to use generative AI, so long as they acknowledge it, how can they say what percentage of text is their own and what percentage is of AI authorship? Who would keep track, and how would one keep track, exactly? Often, what matters in a paper is less the exact text and more the underlying ideas, and how does one count ideas or measure the originality of those ideas? The result may be that, eventually, generative AI takes over the creative part of writing and people feel no more indebted to the generative AI than they do to the computers that perform their data analysis for them. Who even bothers to write in a scientific paper that the data analysis was performed by a computer or that the text was grammar- or spell-checked by software? Over time, it is not difficult to foresee a mindset whereby people view AI-generated as their “own”, much as they view computer-generated data analyses as their own. After all, in each case, the people specify to the machine what it was that they wanted accomplished.

There is a crucial difference—data analyses do, or at least should, yield uniquely correct answers, whereas text written by generative AI represents an infinity of possibilities. But will this difference stop people from using generative AI or coming to view the products as their “own”? I doubt it. As one of my colleagues noted, whether one allows generative AI in one’s classes, students will use it anyway, much as they will use computers to word-process their essays. The only difference will be whether they hide it or not. When one’s grades or job are at stake, people may find themselves believing what it is in their perceived best self-interest to believe. And that point, one may accept whatever creativity one gets because, after all, one believes that it is one’s own.

## 6. Conclusions

The question of whether generative AI is a good thing or a not-so-good thing is an interesting one, but not one that can be properly addressed in this essay. In my own psychology department, some professors encourage the use of generative AI in their courses, others remain on the fence, and still others forbid its use. But forbidding its use may be like fighting against flood waters that have raged out of control because it is not clear how one can know with any confidence whether one’s prohibition has been followed. And as will inevitably follow in such cases, people will take the path of least resistance, especially if others do.

All technologies change the nature of human intelligence and creativity, and what it takes to be adaptable to the environment (Gigerenzer 2022; Sternberg and Preiss 2005). Generative AI is changing, at the very least, our conceptions of what it means to be creative, and it also is changing how many of us will be creative in society. The products we produce may sometimes become more creative and other times less creative. But our human creativity, with such a tool, risks going the way of our penmanship (Heavens 2015; Paoletto 2023), our spelling (Denn 2019), our writing and computation skills (NAEP 2022), and all else that machines have taken away (NAEP 2022; NAEPPLUS+ 2023). It is not clear that this is a loss we should gladly accept, or really accept at all. Paradigm-defying creativity is unlikely to come from AI. So-called Big-C ideas, ones that change the world (Kaufman and Beghetto 2009), are likely to emanate from humans, at least for a long time. But given the many messes the world confronts today, paradigm-defying, Big-C creativity is what the world needs most.
